# Scheduling of a Parcel Delivery System Consisting of an Aerial Drone Interacting with Public Transportation Vehicles

**DOI:** 10.3390/s20072045

**Published:** 2020-04-05

**Authors:** Hailong Huang, Andrey V. Savkin, Chao Huang

**Affiliations:** 1 School of Electrical Engineering and Telecommunications, University of New South Wales, Sydney 2052, Australia; hailong.huang@unsw.edu.au; 2 Electrical Computer and Telecommunications Engineering, University of Wollongong, Wollongong 2500, Australia; ch449@uowmail.edu.au

**Keywords:** UAVs, drones, parcel delivery, scheduling problem, public transportation vehicles

## Abstract

This paper proposes a novel parcel delivery system which consists of a drone and public transportation vehicles such as trains, trams, etc. This system involves two delivery schemes: drone-direct scheme referring to delivering to a customer by a drone directly and drone–vehicle collaborating scheme referring to delivering a customer based on the collaboration of a drone and public transportation vehicles. The fundamental characteristics including the delivery time, energy consumption and battery recharging are modelled, based on which a time-dependent scheduling problem for a single drone is formulated. It is shown to be NP-complete and a dynamic programming-based exact algorithm is presented. Since its computational complexity is exponential with respect to the number of customers, a sub-optimal algorithm is further developed. This algorithm accounts the time for delivery and recharging, and it first schedules the customer which leads to the earliest return. Its computational complexity is also discussed. Moreover, extensive computer simulations are conducted to demonstrate the scheduling performance of the proposed algorithms and the impacts of several key system parameters are investigated.

## 1. Introduction

With the fast development of drones (also called unmanned aerial vehicles (UAVs)) technology, drones have found various applications in civilian domains [1], such as wireless communication support [2], structural health inspection [3], farming [4], surveillance and monitoring [5,6,7] and parcel delivery [8,9]. Thanks to their mobility and flexibility, many logistics companies and many advanced control approaches such as autonomous landing [10], such as Amazon, Alibaba, DHL, SF Express, etc., have started to pay more attention to the application of drones in parcel delivery. Researchers and logistics companies have conducted much research on designing cost-and-time efficient systems such that the delivery can be done in a fast and low-cost way. One model, which has attracted much attention in the research community, is called drone–truck collaboration [11]. Specifically, a truck is equipped with one or several drones. The drones can launch from the truck, deliver to some customers, and then fly to somewhere to dock with the truck. Meanwhile, the truck can service some other customers. Some variants of this model have also been published, such as clustering TSP [12], TSP with drone (TSP-D) [13], minimum cost TSP [14], TSP with Drone Station (TSP-DS) [15], Heterogeneous Delivery Problem (HDP) [16,17], Drone Scheduling Problem (DSP) [18], and Vehicle Routing Problem with Drones (VRPD) [19].

Another model is to equip a depot with a fleet of drones, and the drones fly between customers and the depot [20]. An important problem is the delivery scheduling problem. A number of publications present some theoretical results for this model [21,22]. Comparing to the first model, this model can further reduce the human labour participation. However, the currently available commercial drones are often constrained in flight distance due to the limited on-board battery capacity. For example, Amazon released that their commercial delivery drone can fly about half an hour and travel up to 32 km (https://www.cbsnews.com/news/amazon-unveils-futuristic-plan-delivery-by-drone/). If a customer is located slightly outside the delivery area, they cannot be served by the drones. Therefore, the delivery area of this model is also limited.

Following the second model, some publications have considered the facility location problem under the context of drone delivery to extend the delivery area [23,24]. Clearly, one disadvantage of this method is the investment in constructing the new facilities. The current paper also focuses on addressing this shortcoming. Unlike [23,24] to place more facilities, we introduce the idea of drone–vehicle collaboration [25,26]. Here, the vehicle refers to the public transportation vehicles. Unlike the trucks in the first model, the public transportation vehicles have their own routes and timetables, and they cannot be controlled like the trucks by the logistics companies. The public transportation vehicles can take drones to some places which are unreachable by flying themselves. More importantly, as public transportation vehicles are natural mobile platforms which have already existed in the urban area, this model does not introduce much extra cost.

We propose a novel parcel delivery system involving two delivery schemes. The first one, hereafter called drone-direct scheme, uses a drone to directly fly to the customer and then return to the depot. The second one, hereafter called drone–vehicle scheme, makes use of public transportation vehicles. A drone can travel with a vehicle to some position near the customer like a passenger. Differently, it rests on the roof of the vehicle. Then, the drone leaves the vehicle and flies to the customer. After dropping the parcel, it then flies to another vehicle that can transport it back to the depot. In the two schemes, the drone operates autonomously. Comparing these two schemes, the drone-direct scheme generally achieves fast delivery than the drone–vehicle scheme. However, the former may also consume more energy than the latter, since all the trip is made by a drone itself. In contrast, the drone–vehicle scheme explores the vehicle mobility, which saves the energy for the drone. Thus, after delivery, a drone that follows the drone-direct scheme may need longer time for recharging the battery than the drone–vehicle scheme.

Although the delivery time may be longer as the drone needs to wait for the transportation of the public vehicles, the second scheme creates the possibility of delivering to a customer in a low-cost manner. It is quite useful for a supplier that wishes to cost-effectively deliver to a non-urgent customer. One challenge brought by the drone–vehicle scheme is the collaboration between the drone and the vehicles. With the development of vehicle-to-vehicle communication technology, we believe that in the future a drone can also talk to a normal vehicle to collect the trip information of the vehicle. Then, there will be more options for the drone to travel. In this paper, we limit ourselves to a situation where the vehicle-to-vehicle communication technology is not used, and the drone only travels with public vehicles, whose trip information can be well managed.

We study the fundamental characteristics of the two schemes and also investigate how to select the delivery scheme. Based on these models, we furthermore study the scheduling task for a single drone given a set of customers such that the total delivery time is minimized. This problem has practical applications since many suppliers wish to service their customers in queue in the fastest manner. The time needed to recharge the battery is accounted in our model. In particular, to deliver to a certain customer, the residual energy of the battery has to be greater than that to be consumed. Otherwise, the drone has to recharge the battery before departure. In our formulation, the charging rate can be adjusted, and it covers the case where battery charging is replaced by battery swapping by simply setting the charging rate sufficiently large. With the energy constraint, a scheduling problem is formulated. The solution of the scheduling problem involves the permutation of the to-be-served customers, the time for recharging, and the delivery scheme for each customer. Since the proposed delivery system involves two delivery schemes and if the drone–vehicle scheme is adopted for a certain customer, the trip time further depends on the departure instant. We mathematically formulate this time-dependent scheduling problem and show that it is NP-complete.

To find the optimal solution, we develop a dynamic programming-based algorithm. It gives the optimal schedule, but its computational complexity is exponential with respect to the number of customers, which is infeasible for a slightly higher number of customers. Considering this, an algorithm which finds a sub-optimal solution is developed. This algorithm accounts the time for delivery and recharging and it first schedules the customer which leads to the earliest return. It is shown that the sub-optimal algorithm is much more computationally efficient than the exact algorithm. The performance gap between them in terms of the total time to complete all the deliveries is demonstrated via extensive computer simulations. The simulation results display that the sub-optimal algorithm achieves close performance to the exact algorithm. Finally, we conduct more simulations to investigate the impacts of some important system parameters including the energy consumption rate, recharging rate, the timetable of the vehicles, and the speed of the drone.

The rest of the paper is organized as follows. Section 2 discusses some related publications. Section 3 presents the system models and states the studied problem. Section 4 presents the proposed solutions. In Section 5, extensive simulation results are provided and Section 6 concludes the paper with some discussions about future research directions.

## 2. Related Work

The current paper considers the drone delivery application and the problem under investigation is, in essence, a scheduling problem. The scheduling problem is a conventional problem, which in general consists of the allocation of resources over time to perform a set of jobs. The aim of scheduling is to figure out the optimal schedule, among all feasible schedules, which optimizes a given criterion function. In this paper, the drone is the resource, delivering a customer is a job, and the aim is to minimize the total time for delivery.

In terms of drone delivery, the first category of relevant publications is more or less related to the conventional travelling salesman problem (TSP). The papers [11,13,15,16,17,19] consider the scenario when a truck carries one or several drones and investigate how to schedule the deliveries for both the truck and the drones. A very basic idea is presented in [11] which firstly constructs a TSP tour for the truck with all the customers. Then, it considers whether assigning some customer to a drone reduces the total delivery time. The reference [12] presents a method which clusters the customers first and then constructs a TSP tour. The authors of [13] discuss a variant scenario of [11], where the drone also needs to follow the road network like trucks. This helps the drone to avoid collisions with buildings in urban environments, however, it loses the benefits of faster delivery by the drone. Instead of the total delivery time, the reference [14] aims at minimizing the cost associated with the deliveries, where the transportation cost of truck and drone and the part relating to the time a vehicle has to wait for the other are accounted. The paper [15] presents a model involving a drone station, which stores a fleet of drones and is activated by a truck when the truck loads parcels to the station. This paper investigates the problem of customer assignment with the objective to minimize the total delivery time. Different from the above models, in [16] the truck is used only to transport a drone and it does not deliver to customers. The authors model it as a generalized TSP and the solution includes not only the route of the truck but also where the drone launches and returns to the truck. The paper [17] considers a similar scenario to [16], while the authors further account the delivery order constraint (i.e., some customers should be served before some others). They show the NP-hardness of the problem and derive the lower bound of the optimal time to deliver to all the customers. Heuristic algorithms are also provided to address the problem. The paper [18] also considers the same scenario, while it assumes the route of the truck is fixed and discusses how to schedule multiple drones. The authors of [19] formulate the Vehicle Routing Problem with Drones problem (VRPD) as a mixed integer programming model and propose a branch-and-price algorithm to address it. In particular, a drone is allowed to deliver more than one customers in one trip. Considering this, many routing methods can be applied if the drone can deliver multiple parcels in one trip with the presence of charging static or mobile stations [27,28].

The second category of related work is about the drone-direct scheme, where only drones are used for delivery. The paper [20] considers the scenario where a drone delivers more than one customers in a trip, although this is not the favoured mode by logistics companies. Clearly, if there is only one drone, the problem is a variant of TSP; and if there are multiple drones, the problem turns to be a variant of vehicle routing problem (VRP), which is also a variant of TSP. In [20], the energy constraint of drones is accounted, and the authors investigate the influence of the payload on the energy consumption. In [21], the authors analyze the system stability based on the queuing theory. The paper [22] investigates the impact of the unreliability of drones on the delivery performance due to inaccurate estimation on the battery consumption rate. Since the capacity of the on-board battery is often constrained, the delivery range by the drone-direct scheme is limited. The paper [23] proposes a model to deploy a certain number of charging stations such that the coverage of customers is maximized, which is a coverage problem. In this paper, a drone only delivers to one customer and the impact of the payload is also accounted. The reference [24] addresses the problem of positioning warehouses as well as refuel stations.

The last category is about the utilization of public transportation vehicles [25,26]. Unlike trucks in the first category, the public transportation vehicles are not controlled by logistics companies. The authors of [25] carried out interviews with experts from relevant industries and concluded that the utilization of autonomous mobility is a feasible alternative delivery method. The reference [26] presents an idea of using public transportation vehicles to move drones to some positions of interest and even recharge the drones.

There are some similarities and differences between the current paper and the aforementioned ones. The model proposed herein combines the drone-direct scheme and the drone–vehicle scheme. It involves the delivery scheme selection which depends on the location of a specific customer, the residual energy of the drone, and the timetable of the public transportation vehicles. This paper targets the scheduling problem and the time for recharging is accounted. The aforementioned references, especially those in the first category, generally do not consider this, whereas they assume that a sufficient number of batteries is available at the truck and the battery can be replaced with a fresh one after each delivery.

## 3. System Model and Problem Statement

**Figure 1:**
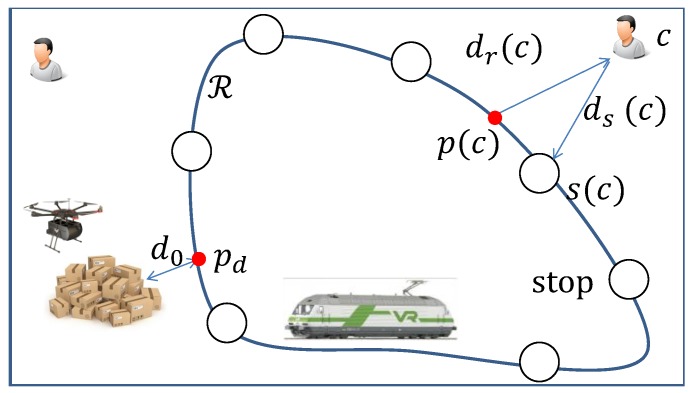
The considered parcel delivery system.

Consider a parcel delivery system consisting of a depot, a drone and a fleet of public transportation vehicles. The vehicles keep running on a predefined route R following a timetable T (Notice that this paper only considers the public transportation vehicles, such as trains and trams, whose timetables are accurate. The impacts of the stochastics of unreliable (in terms of timetable) public transportation vehicles such as buses and environmental factors such as wind are left as our future work.). Along the route R, there is a set of stops S. There are two delivery schemes: drone-direct delivery and drone–vehicle delivery. In the drone-direct delivery scheme, a drone departs the depot and flies to the customer directly. After dropping the parcel, it returns to the depot. In the drone–vehicle delivery scheme, after departing the depot, a drone catches a vehicle and travels with the vehicle on the roof. The drone leaves the vehicle at some position near the customer. After finishing the delivery, the drone flies to the nearest vehicle stop s∈S and waits for a vehicle that can transport it back to the depot. The current paper considers a scenario where the vehicle route is closed, see Figure 1. Notice that the discussed route can be extended to the complex public transportation network and as will be shown later, a delivery time calculator is needed to compute the delivery time between the depot and a customer in the complex public transportation network. The main notations and their meanings are listed in Table 1.

The drone-direct scheme is easy to implement and fast to complete a delivery. However, a shortcoming is the limitation in delivery range due to the constrained on-board battery capacity. The drone–vehicle scheme overcomes this drawback thanks to the transportation provided by vehicles. A vehicle can take a drone to the position near the customer and such a position can be outside the direct flight range of a drone. Clearly, a negative point of the drone–vehicle scheme is that it may take a long time, since the drone may wait to catch vehicles.

We assume that the drone flies at a constant speed *v* and the energy consumption rate *p* is also a constant when it flies. When the drone travels with vehicles, it turns off the motors. Thus, we assume that the energy consumption during this time is zero, since it is negligible compared to that consumed by flying. We also assume that the battery recharging rate *q* is a constant. The value of *q* depends on the type of drones. For example, charging DJI Spark directly with USB takes 80 min. Notice that the used energy consumption and recharging models can be extended to some more accurate models. When a drone is at the depot, it recharges the battery up to $E_{0}$, which represents the battery capacity. A drone can be sent to deliver only if the residual energy is no less than the energy to be consumed for the delivery plus a safety residual energy ϵ.

**Figure 2:**
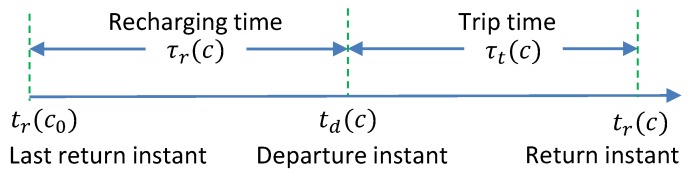
Illustration of key notations.

No matter which delivery scheme is used, after returning from the last customer $c_{0}$ at time instant $t_{r}\left(c_{0}\right)$, the drone may start to recharge the battery. The recharging time is denoted by $\tau_{r}\left(c\right)$ if the next customer to be delivered is *c*. Once the energy is sufficient, the drone may depart to deliver to customer *c* at instant $t_{d}\left(c\right)$. The return instant for customer *c* is denoted by $t_{r}\left(c\right)$ and the trip time is $\tau_{t}\left(c\right)=t_{r}\left(c\right)-t_{d}\left(c\right)$. Throughout this paper the notation *t* with some subscript or superscript is used to represent a time instant, while τ is used to represent a time interval. The relations between some important notations are demonstrated in Figure 2.

The two delivery schemes both have some limitations in terms of delivery area. For the drone-direct scheme, its delivery area is simply a circular area, denoted by $A_{1}$. Let $d_{d}\left(c\right)$ be the distance between customer *c* and the depot and let $\tau_{f}^{dd}\left(c\right)$ denote the flight time to deliver to customer *c*. The drone takes off from the depot, flies to the customer, drops the parcel, returns to the depot and lands. We assume that the drone flies at a pre-defined altitude. The take-off time from the ground to this given altitude is denoted by $\delta_{1}$ and that for landing is denoted by $\delta_{2}$. Notice that the flight time equals the trip time mentioned above plus the time for take-off and landing:

(1) $$\tau_{t}^{dd}\left(c\right)=\tau_{f}^{dd}\left(c\right)=\frac{2d_{d}\left(c\right)}{v}+\delta_{1}+\delta_{2}.$$

Here, the superscript dd is used to indicate the drone-direct scheme. Below, we will use the superscript dv to indicate the drone–vehicle scheme. We have the following constraint:

(2) $$\frac{2d_{d}\left(c\right)}{v}p\leq E_{0}-\epsilon -\epsilon_{1}-\epsilon_{2},$$

where $\epsilon_{1}$ and $\epsilon_{2}$ and are the energy consumption for take-off and landing, respectively. From (2) we obtain:

(3) $$d_{d}\left(c\right)\leq \frac{E_{0}-\epsilon -\epsilon_{1}-\epsilon_{2}}{2p}v,$$

i.e., if the constraint (3) holds for customer *c*, we say customer *c* is within the delivery area $A_{1}$.

To deliver to a customer *c*, there is a requirement of the residual energy of the drone, i.e., it should be no less than the to be consumed energy for *c* plus the safety residual energy ϵ. When the drone returns from customer $c_{0}$, if the residual energy *E* is not enough to deliver to customer *c*, it needs to recharge the battery. Let $\tau_{r}^{dd}\left(c\right)\geq 0$ denote the recharging time for *c*, and it should satisfy the following constraint:

(4) $$E+\tau_{r}^{dd}\left(c\right)q-\tau_{f}^{dd}\left(c\right)p\geq \epsilon +\epsilon_{1}+\epsilon_{2},$$

from which we obtain:

(5) $$\tau_{r}^{dd}\left(c\right)=\max\left\{0,\frac{\epsilon +\epsilon_{1}+\epsilon_{2}-E+\tau_{f}^{dd}\left(c\right)p}{q}\right\}.$$

In words, if the residual energy is sufficient to delivery customer *c*, the drone can depart immediately without recharging; otherwise, it has to recharge for a minimum time $\tau_{r}^{dd}\left(c\right)$. With the recharging time and the above trip time, the return instant after delivering customer *c*, i.e., $t_{r}^{dd}\left(c\right)$, is given by:

(6) $$t_{r}^{dd}\left(c\right)=t_{r}\left(c_{0}\right)+\tau_{r}^{dd}\left(c\right)+\tau_{t}^{dd}\left(c\right).$$

For the drone–vehicle scheme, the delivery area $A_{2}$ further depends on the route R. The trip time is no smaller than the flight time for customer *c*, i.e., $\tau_{t}^{dv}\left(c\right)\geq \tau_{f}^{dv}\left(c\right)$, since the drone travels with vehicles in this scheme. For this scheme, we introduce some new notations. Let $p_{d}$ be the point on R which is the closest to the depot. Let $d_{0}$ be the distance between the depot and $p_{d}$, see Figure 1. For customer *c*, let p(c) denote the point on R, which is the closest to the customer *c*. Let $d_{r}\left(c\right)$ denote the distance between the customer *c* and the point p(c). Let s(c)∈S denote the stop which is the closest to customer *c* and let $d_{s}\left(c\right)$ denote the distance between customer *c* and stop s(c). The flight time $\tau_{f}^{dv}\left(c\right)$ for customer *c* is defined as follows and if it satisfies the right inequality, customer *c* can be delivered by the drone–vehicle scheme:

(7) $$\tau_{f}^{dv}\left(c\right)=\frac{d_{0}+d_{r}\left(c\right)+d_{s}\left(c\right)+d_{0}}{v}+\delta_{1}+\delta_{2}.$$

We have the constraint on the energy expenditure:

(8) $$\frac{d_{0}+d_{r}\left(c\right)+d_{s}\left(c\right)+d_{0}}{v}p\leq E_{0}-\epsilon -\epsilon_{1}-\epsilon_{2},$$

from which we can obtain:

(9) $$d_{r}\left(c\right)+d_{s}\left(c\right)\leq \frac{E_{0}-\epsilon -\epsilon_{1}-\epsilon_{2}}{p}v-2d_{0}.$$

If the customer *c* satisfies the constraint (9), we say this customer locates within $A_{2}$. There are two assumptions regarding the energy consumption. One is that the time needed to drop off the parcel is negligible. The other is that we assume that the stops and the point $p_{d}$ are with some platforms where the drone can rest. Thus, the corresponding energy consumption when the drone is resting is ignored. More information about the second assumption will be given later in this section. Similar to (5), the time of recharging to deliver to customer *c* is given as follows:

(10) $$\tau_{r}^{dv}\left(c\right)=\max\left\{0,\frac{\epsilon +\epsilon_{1}+\epsilon_{2}-E+\tau_{f}^{dv}\left(c\right)p}{q}\right\}.$$

**Figure 3:**
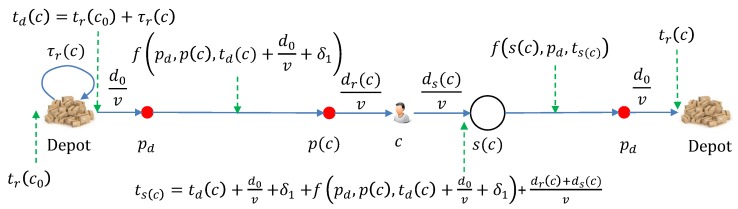
An illustration to compute the several key time instants and intervals for the drone–vehicle scheme. Here the superscript dv is not displayed as this only suits the drone–vehicle scheme.

**Figure 4:**
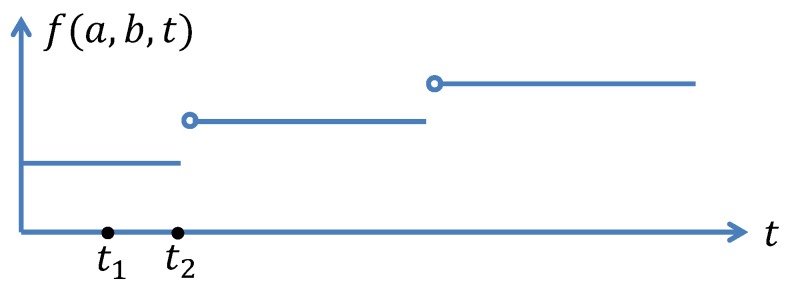
An example of function f(a,b,t).

Now, we consider the model of return instant for the drone–vehicle scheme, see Figure 3. Let $c_{0}$ denote the last delivered customer. After recharging the battery for $\tau_{r}^{dv}\left(c\right)$, the drone departs at instant $t_{d}^{dv}\left(c\right)=t_{r}\left(c_{0}\right)+\tau_{r}^{dv}\left(c\right)$. The drone then arrives at the point $p_{d}$, where it lands on the platform to rest and waits for the coming vehicle. The time needed to reach $p_{d}$ is given by $\frac{d_{0}}{v}+\delta_{1}$ and the arrival instant is $t_{d}^{dv}\left(c\right)+\frac{d_{0}}{v}+\delta_{1}$. Introduce a function f(a,b,t) which computes the minimum time needed for a considered vehicle to travel from point *a* to point *b* on route R following the timetable T, when the drone arrives at *a* at time instant *t*. Considering the basics of the timetable T, the function f(a,b,t) is a multi-step function and the duration for each step and the gap between two consecutive steps may vary since the timetable T may be time-dependent. An illustration is shown in Figure 4. Clearly, arriving $p_{d}$ at time instants $t_{1}$ and $t_{2}$ result in the same time to reach p(c). This is because that the vehicle arrives at $p_{d}$ at time instant $t_{2}$. If the drone arrives at $p_{d}$ later than $t_{2}$, it just misses the vehicle and has to wait for the next. When the drone arrives at $t_{1}$, it has to wait for a duration $t_{2}-t_{1}$. Obviously, such a waiting may be unnecessary for the drone–vehicle scheme, as the drone can further recharge the battery for some time. Thus, we assume that the timetable T is available to the drone and the drone can depart the depot at an instant such that it arrives at $p_{d}$ at the same with a vehicle. To achieve this, we introduce another function g(t) which computes the time for vehicles to arrive at $p_{d}$ from their current positions, and this computation is done at instant *t*. Here, we need to do the computation once the drone is ready to depart, i.e., at instant $t_{d}^{dv}\left(c\right)$. As there are several vehicles running on the route at the same time, the function $g(t_{d}^{dv}\left(c\right))$ returns a positive vector whose size equals the number of vehicles. We only consider the one which is the most closest to $\frac{d_{0}}{v}$ and no smaller than $\frac{d_{0}}{v}$. For those which are smaller than $\frac{d_{0}}{v}$, the drone will never catch the corresponding vehicles. We denote such a value by $\bar{g}\left(t_{d}^{dv}\left(c\right)\right)$. Then, the drone can recharge the battery at the depot for an extra period of $\bar{g}\left(t_{d}^{dv}\left(c\right)\right)-\frac{d_{0}}{v}-\delta_{1}$. Of course, arrival time at p(c) will remain the same, since the waiting time at $p_{d}$ is simply shifted to the depot. The further recharged energy may reduce the time in the following recharging process.

The arrival time at $p_{d}$ can be still regarded as $t_{d}^{dv}\left(c\right)+\frac{d_{0}}{v}$. Then, the travel time from $p_{d}$ to p(c) is given by $f(p_{d},p\left(c\right),t_{d}^{dv}\left(c\right)+\frac{d_{0}}{v}+\delta_{1})$. After that, the drone leaves the vehicle at point p(c), flies to the customer *c*, drops off the parcel, and then flies to the stop s(c). When the drone arrives at s(c), the time instant, denoted by $t_{s(c)}$, is computed as follows:

(11) $$t_{s(c)}=t_{d}^{dv}\left(c\right)+\frac{d_{0}}{v}+\delta_{1}+f\left(p_{d},p\left(c\right),t_{d}^{dv}\left(c\right)+\frac{d_{0}}{v}+\delta_{1}\right)+\frac{d_{r}\left(c\right)+d_{s}\left(c\right)}{v}.$$

Then, from s(c) to $p_{d}$, the travel time is $f(s\left(c\right),p_{d},t_{s(c)})$, which is followed by the final flight from $p_{d}$ to the depot. Therefore, the return instant $t_{r}^{dv}\left(c\right)$ is as follows:

(12) $$t_{r}^{dv}\left(c\right)=t_{s(c)}+f\left(s\left(c\right),p_{d},t_{s(c)}\right)+\frac{d_{0}}{v}+\delta_{2}.$$

The trip time is:

(13) $$\tau_{t}^{dv}\left(c\right)=t_{r}^{dv}\left(c\right)-t_{d}^{dv}\left(c\right).$$

**Remark** **1.** *Notice that for a given timetable of vehicles running on a simple route like the currently considered closed route, the functions f(), g(), and $\bar{g}\left(\right)$ can be obtained easily. The whole picture of drone parcel delivery would be that one depot is associated with a fleet of drones and the drones can reach customers and return to the depot via multiple public transportation vehicles. Specifically, a drone can transfer between vehicles like passengers to reach its destination. In this case, these may be multiple positions like $p_{d}$ in Figure 1, where the drone can join and leave the transportation network. We will meet the problem of how to estimate the time and energy cost for delivering a customer. In [29], we have developed a one-way trip planning algorithm to plan the path from the depot to a customer for a drone within a given deadline. We will improve it and develop a round-trip planning method. With this technique, we will further consider the scheduling problem in the whole picture: scheduling deliveries with multiple drones and multiple customers either directly or via the public transportation network.*

**Remark** **2.** *We also note that the above models ignore the impact of package weight. In practice, the heavier the package, the larger the energy consumption rate. To accommodate this feature, we can extend the current models by introducing another notation, i.e., w(c), representing the energy consumption rate for delivering customer c. Note that the function w(c) depends on the package weight of customer c, and this function can be known by carrying out real experiments. With the function w(), the energy consumption models for both delivery schemes are modified as follows. Specifically, constraint (2) becomes:*

(14) $$\frac{d_{d}\left(c\right)}{v}\left(w\left(c\right)+p\right)\leq E_{0}-\epsilon -\epsilon_{1}-\epsilon_{2}.$$

*Constraint (8) becomes:*

(15) $$\frac{d_{0}+d_{r}\left(c\right)}{v}w\left(c\right)+\frac{d_{s}\left(c\right)+d_{0}}{v}p\leq E_{0}-\epsilon -\epsilon_{1}-\epsilon_{2},$$

*Other models for these two schemes can be modified accordingly.*

So far, the recharging time, flight time, and trip time have been discussed for both schemes, respectively. If $c\in A_{1}\cap A_{2}$, we need to make a decision on the selection of the delivery schemes. Introduce a binary variable x(c) signifying the selected scheme:

(16) $$x\left(c\right)=\left\{\begin{array}{c} \begin{array}{c} 1,\mathrm{if}\;c\;\mathrm{is}\;\mathrm{served}\;\mathrm{by}\;\mathrm{drone}-\mathrm{direct}\;\mathrm{scheme};\\ 0,\mathrm{otherwise}. \end{array} \end{array}\right.$$

Then, the return instant for delivering customer *c*, the flight time and the trip time can be represented as follows:

(17) $$t_{r}\left(c\right)=x\left(c\right)t_{r}^{dd}\left(c\right)+\left(1-x\left(c\right)\right)t_{r}^{dv}\left(c\right),$$

(18) $$\tau_{f}\left(c\right)=x\left(c\right)\tau_{f}^{dd}\left(c\right)+\left(1-x\left(c\right)\right)\tau_{f}^{dv}\left(c\right),$$

(19) $$\tau_{t}\left(c\right)=x\left(c\right)\tau_{t}^{dd}\left(c\right)+\left(1-x\left(c\right)\right)\tau_{t}^{dv}\left(c\right).$$

Consider a set of customers C in the area $A_{1}\cup A_{2}$ (|C|=n). Given the timetable T, the functions of f() and g() are known. Let λ denote a schedule containing the permutation $\left\{c_{1},c_{2},\ldots ,c_{n}\right\}$, the recharging times $\left\{\tau_{r}\left(c_{1}\right),\tau_{r}\left(c_{2}\right),\ldots ,\tau_{r}\left(c_{n}\right)\right\}$, and the selection of the delivery scheme $\left\{x\left(c_{1}\right),x\left(c_{2}\right),\ldots ,x\left(c_{n}\right)\right\}$. Clearly, for any customer *c*, both the return instant $t_{r}\left(c\right)$ and energy consumption (which is proportional to $\tau_{f}\left(c\right)$ depend only on $\tau_{r}\left(c\right)$ and x(c). In other words, with the basic information in λ, the return instant and the corresponding energy consumption can be further computed. Specifically, the initial energy is $E_{0}$ (the initial recharging is not necessary, i.e., $\tau_{r}\left(c_{1}\right)=0$, since the battery is full) and let *E* represent the residual energy after delivering a customer. The energy consumed for serving customer $c_{1}$ is given by $p\tau_{f}\left(c_{1}\right)$. The recharging time for customer $c_{2}$ is given by $\tau_{r}\left(c_{2}\right)$. Thus, the residual energy of the drone just before the departure for customer $c_{2}$ is given by $\min\{E_{0},E+q\tau_{r}\left(c_{2}\right)-p\tau_{f}\left(c_{1}\right)\}$. It is easy to compute the residual energy of the drone before the departure for any customer $c_{j}$ (2≤j≤n) in the similar way, i.e., $\min\{E_{0},E+q\sum_{i=1}^{j}\tau_{r}\left(c_{i}\right)-p\sum_{i=1}^{j-1}\tau_{f}\left(c_{i}\right)\}$.

The problem under consideration is to figure out the optimal schedule λ such that the return instant after delivering the final customer is minimized. Let $c_{0}$ be a virtual customer and $t_{r}\left(c_{0}\right)=0$, or we say the starting instant is zero. Then, the return instant from $c_{1}$ is given by $t_{r}\left(c_{1}\right)=t_{r}\left(c_{0}\right)+\tau_{r}\left(c_{1}\right)+\tau_{t}\left(c_{1}\right)$. The return instant from $c_{2}$ is given by $t_{r}\left(c_{2}\right)=t_{r}\left(c_{1}\right)+\tau_{r}\left(c_{2}\right)+\tau_{t}\left(c_{2}\right)$. From them, we obtain $t_{r}\left(c_{2}\right)=t_{r}\left(c_{0}\right)+\sum_{i=1}^{2}\tau_{r}\left(c_{i}\right)+\sum_{i=1}^{2}\tau_{t}\left(c_{i}\right)$. Then, the return instant from $c_{n}$ is $t_{r}\left(c_{n}\right)=t_{r}\left(c_{0}\right)+\sum_{i=1}^{n}\tau_{r}\left(c_{i}\right)+\sum_{i=1}^{n}\tau_{t}\left(c_{i}\right)$.

The problem of minimizing the final return instant is formulated as follows:

(20) $$\min\;\sum_{i=1}^{n}\left(\tau_{r}\left(c_{i}\right)+\tau_{t}\left(c_{i}\right)\right)=\min\;\sum_{i=1}^{n}\left(\tau_{r}\left(c_{i}\right)+x\left(c_{i}\right)\tau_{t}^{dd}\left(c_{i}\right)+\left(1-x\left(c_{i}\right)\right)\tau_{t}^{dv}\left(c_{i}\right)\right)$$

subject to

(21) $$\begin{array}{c} x\left(c_{j}\right)t_{r}^{dd}\left(c_{j}\right)+\left(1-x\left(c_{j}\right)\right)t_{r}^{dv}\left(c_{j}\right)-\tau_{r}\left(c_{j}\right)>\\ x\left(c_{j-1}\right)t_{r}^{dd}\left(c_{j-1}\right)+\left(1-x\left(c_{j-1}\right)\right)t_{r}^{dv}\left(c_{j-1}\right),\;\forall 1\leq j\leq n, \end{array}$$

(22) $$\begin{array}{c} \min\{E_{0},E+q\sum_{i=1}^{j}\tau_{r}\left(c_{i}\right)-p\sum_{i=1}^{j-1}\left(x\left(c_{i}\right)\tau_{f}^{dd}\left(c_{i}\right)+\left(1-x\left(c_{i}\right)\right)\tau_{f}^{dv}\left(c_{i}\right)\right)\}-\\ p(x\left(c_{j}\right)\tau_{f}^{dd}\left(c_{j}\right)+\left(1-x\left(c_{j}\right)\right)\tau_{f}^{dv}\left(c_{j}\right))\geq \epsilon ,\forall 2\leq j\leq n. \end{array}$$

The variables of this problem include the delivery sequence $c_{1},\ldots ,c_{n}$, the charging times $\tau_{r}\left(c_{1}\right),\ldots ,\tau_{r}\left(c_{n}\right)$ and the delivery scheme selections $x\left(c_{1}\right),\ldots ,x\left(c_{n}\right)$. The constraints (21) require that the return instant for delivering a customer should always be greater than that for the last delivered customer by the recharging time. The constraints (22) require that for any customer $c_{j}$ (2≤j≤n), the residual energy after delivering customer $c_{j}$ no less than ϵ. Notice that for the customer $c_{1}$, $E_{0}-p\tau_{f}\left(c_{1}\right)\geq \epsilon$ holds as $c_{1}\in C$ is inside the area $A_{1}\cup A_{2}$.

The analysis of the problem complexity is based on the relaxation of the original problem to a problem with known complexity through the following assumptions:

- Only the drone–vehicle scheme is adopted;
- The energy constraints (22) are removed;
- The timetable T does not vary with time, i.e., the vehicles keep running on the route periodically.

With the first assumption, the issue of scheme selection is not involved any more and the problem turns to be a conventional scheduling problem, where delivering a customer can be regarded as a job and the recharging time plus the flight time can be regarded as the processing time. With the second assumption, two jobs can be processed without idle time in between. With the third assumption, the functions f() and g() are not time varying. With these assumptions, the original problem (20)–(22) is reduced to the scheduling problem to minimize the makespan with multi-step deteriorating time on a single machine, which is NP-complete [30]. Obviously, without these assumptions, the original problem is much more complex. It is costly to address the considered problem optimally due to its NP-hardness.

## 4. Proposed Solution

In this section, we discuss how to address the original problem (20). We first present a dynamic programming-based exact algorithm that can figure out the optimal schedule, and then we develop a sub-optimal algorithm.

### 4.1. Dynamic Programming-Based Exact Algorithm

Consider a subset of customers M⊆C and a customer c∈M. Denote by T(M,c) the earliest return instant to deliver to all the customers in M with *c* as the final delivered customer. Clearly, if the size of M is 1, i.e., |M|=1, then M={c} and $T\left(M,c\right)=0+\tau_{r}\left(c\right)+\tau_{t}\left(c\right)$, where 0 represents the initial instant. Notice that for the determination of the delivery scheme for each customer, the scheme which makes the drone to return to the depot at the earliest instant is selected. If |M|>1, consider that the drone delivers the customer *c* right after delivering the customer $c_{0}$. The return instant T(M,c) after delivering *c* should equal to the return instant after delivering $c_{0}\in M-\left\{c\right\}$ plus the recharging time and the trip time for *c*, i.e., $\tau_{r}\left(c\right)$ and $\tau_{t}\left(c\right)$. With this idea, T(M,c) can be computed in a standard recursive manner as follows:

(23) $$T\left(M,c\right)=\min_{c_{0}\in M-\left\{c\right\}}\left\{T\left(M-\left\{c\right\},c_{0}\right)+\min_{dd,dv}\left\{\tau_{r}^{dd}\left(c\right)+\tau_{t}^{dd}\left(c\right),\tau_{r}^{dv}\left(c\right)+\tau_{t}^{dv}\left(c\right)\right\}\right\}.$$

Based on (23), the exact algorithm is presented in Algorithm 1. If |M|=1, T(M={c},c) can be computed straightforwardly, i.e., $T\left(M,c\right)=\min_{dd,dv}\left\{\tau_{t}^{dd}\left(c\right),\tau_{t}^{dv}\left(c\right)\right\}$. Here, as this is the first delivery, the drone does not need to recharge. So, both $\tau_{r}^{dd}\left(c\right)$ and $\tau_{r}^{dv}\left(c\right)$ are 0. With all of T(M={c},c) pre-computed, the exact algorithm checks M of size 2, 3, and up to *n*. For each size, the algorithm figures out the best last second customer $c_{0}$ such that T(M,c) is minimized. Obviously, to achieve this, the already computed $T(M-\left\{c\right\},c_{0})$ ($\forall c_{0}\in M-\left\{c\right\}$) is used. Moreover, in Line 4, the algorithm selects the delivery scheme by comparing $\tau_{r}^{dd}\left(c\right)+\tau_{t}^{dd}\left(c\right)$ and $\tau_{r}^{dv}\left(c\right)+\tau_{t}^{dv}\left(c\right)$, and the smaller one is selected. Notice that, the calculations of $\tau_{r}^{dd}\left(c\right)$, $\tau_{t}^{dd}\left(c\right)$, $\tau_{r}^{dv}\left(c\right)$, and $\tau_{t}^{dv}\left(c\right)$ all depend on the return instant of the last visited customer, i.e., $c_{0}$ in Line 4. The residual energy *E* should be updated in the similar way as T(M,c) to assist these computations.
**Algorithm 1:** The exact algorithm.
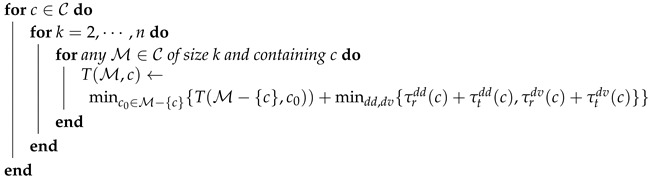


**Optimality**: We first state that there may exist multiple optimal solutions to the considered problem. It is easy to understand that in some extreme cases where all the customers are inside the delivery range of the drone and using drone-direct scheme leads to shorter delivery time for all these customers, the drone–vehicle scheme will not be used for any customers. Then, the drone should depart with the energy of $E_{0}$ and when it returns the residual energy should be ϵ, since this is the best way to save time on recharging. The final return instant is independent on the customer permutation, if the drone recharges the battery for a just sufficient time to deliver the next customer. The existence of multiple optimal solutions also holds when the drone–vehicle scheme is used. For example, in the case where two customers locate on two sides of the vehicle route and they have the same point on the route for the drone to leave the vehicle and the same drone returning stop. Then, delivering these two customers in any order leads to the same trip time and recharging time.

We now state that the solution obtained by Algorithm 1, i.e., including the permutation $\left\{c_{1},c_{2},\ldots ,c_{n}\right\}$, the recharging times $\left\{\tau_{r}\left(c_{1}\right),\tau_{r}\left(c_{2}\right),\ldots ,\tau_{r}\left(c_{n}\right)\right\}$, and the selection of the delivery scheme $\left\{x\left(c_{1}\right),x\left(c_{2}\right),\ldots ,x\left(c_{n}\right)\right\}$, is optimal in terms of the overall delivery time. In Algorithm 1, we want to find out the last customer to be delivered among the set of C, such that the overall delivery time is the shortest. This time is stored in T(C,c), if *c* is the last customer. To achieve this, we check all the customers in the set of C, see Line 1. Since *c* is the last customer to be delivered, the overall delivery time should be the sum of the return instant from the n−1th customer ($T(C-\left\{c\right\},c_{0})$) and the recharging and trip time for the *n*th customer *c*, see Line 4. Then, we need to know what is the return instant to deliver n−1 customers. Thus, for a customer *c* as the last customer, we need to know the return instant to deliver n−1,n−2,…,2 customers, and we can call them in a recursive manner following (23). For implementation, we compute them in a forward way (see Lines 2–6), since it is impossible to compute the return instant to deliver to a larger customer set before the completion of its subsets. After these computations, we can have the smallest return instant T(C,c), where *c* is the last customer. As there are *n* selections for the last customer, we pick the smallest one among *n* values of T(C,c), and this gives the shortest delivery time. Also, the last customer is known, and by a standard backtracking algorithm, the permutation, the recharging time, and the delivery scheme selection can be computed easily.

**Complexity**: we also analyze the complexity of this algorithm. For *n* customers, there are O(n) choices for the customer *c*. For a certain customer *c* and given that $\forall c_{0}\in M-\left\{c\right\}$, $T(M-\left\{c\right\},c_{0})$ has been computed already, for each subset M, the complexity of (23) is O(|M|) and the worst case is O(n). In the dynamic programming term, solving (23) is solving a subproblem. Thus, we need to know how many subproblems to be solved, which is consistent with the number of subsets we need to generate. Clearly, there are $C_{n-1}^{k-1}$ subsets having customer *c* and with the size of *k* (here the notation $C_{n-1}^{k-1}$ represents the combination number). Then, for *n* customers, the total number of subproblems is $O\left(\sum_{k=1}^{n-1}C_{n-1}^{k-1}\right)=O\left(2^{n}\right)$. Therefore, the overall complexity of the exact algorithm is $O(n^{2}2^{n})$. This is much less than that of the naive algorithm which evaluates all the permutations of customers, i.e., O(n!). However, it is still infeasible for a slightly higher number of customers, because both the time complexity as well as the space required (to store T(M,c) and the corresponding residual energy) are exponential with respect to *n*. As a consequence, it is reasonable to design a heuristic algorithm to find sub-optimal solutions, which should be computationally efficient and applicable to large scale cases.

### 4.2. Sub-Optimal Algorithm

Considering the weakness of the exact algorithm in scaling, this subsection presents a sub-optimal algorithm which can quickly finds a feasible solution to the scheduling problem (20). The basic idea of the sub-optimal algorithm is to first schedule the customer which enables the earliest return and we order the customers iteratively. Specifically, when we consider whether to put a customer *c* in the queue, we compute the time needed to recharge the battery upon the remaining energy. As the recharging time relates to the flight time which does not depend on the departure instant (see (2) and (8)), the recharging times for all the remaining customers can be computed. Then, the corresponding departure instant is known for each customer, and the return instants can be calculated further. The customer with the earliest return instant (departure instant plus the recharging time and the trip time) is scheduled.

As shown in Algorithm 2, in Line 1, the initial return instant $t_{r}$ is set as 0 and the initial energy is $E_{0}$. When there are some customers unscheduled in the set C, for a customer *c*, the corresponding recharging time and trip time for the two delivery schemes can be computed according to (2), (5), (10) and (13). With these values, the return instants for the two delivery schemes are obtained (Line 4 and 5). Furthermore, it makes a decision between the two delivery schemes by selecting the one that leads to the earliest return instant for customer *c* (Line 6). After checking all the remaining customers in C, the customer which has the earliest return instant is found out, see Line 8. By backtracking, the delivery scheme for this customer can be known. Then, in Line 9, the residual energy is updated based on the selected customer to be delivered *c* and it is also removed from the set C. The scheduling process terminates when all the customers are scheduled.

**Complexity**: the computational complexity of this sup-optimal algorithm is only $O(n^{2})$, where O(n) is for the while-loop and O(n) is for the for-loop, which is much less than that of the exact algorithm. However, this algorithm generally cannot give the optimal solution. The approximation factor of this algorithm to the exact algorithm depends on specific cases and in the next section, we will demonstrate the performance gap between them via computer simulations.
**Algorithm 2:** The sub-optimal algorithm.
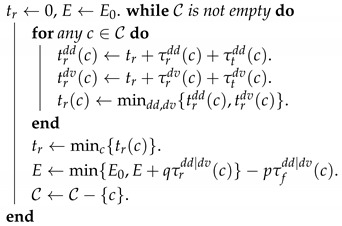


## 5. Simulation Results

In this section, the results of computer simulations, conducted by Matlab, are presented to demonstrate the performance of the sub-optimal algorithm against the exact algorithm. The parameter turnings are investigated as well.

**Figure 5:**
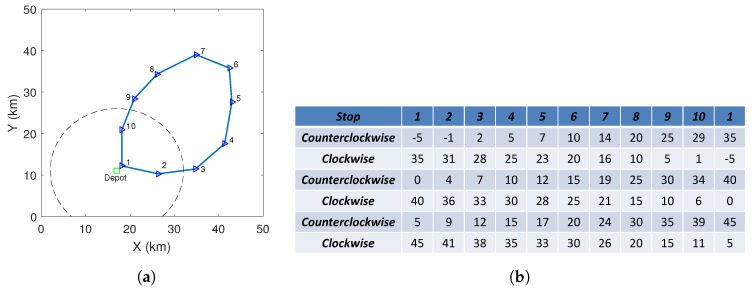
(**a**) The considered area with a circular trajectory. (**b**) Timetable T (in minutes).

In an area as shown in Figure 5a, some vehicles keep running on the given route. There are 10 stops along this route where the drone can rest (represented by triangles with labels). There is a timetable T associated with these stops, see Figure 5b where the unit is minute. The vehicles are running in two directions: clockwise and counterclockwise. We generate the basis of the timetable which is the middle two rows in Figure 5b. Specifically, at instant 0, there is a vehicle departs stop 1, it moves counterclockwise and arrives at stop 2 at instant 4, stop 3 at instant 7, etc. This vehicle finally returns to stop 1 via stop 10 and the arrival time at stop 1 is at instant 40. At instant 0, another vehicle departs stop 1 and it moves clockwise. It arrives at stop 10 at instant 6 and at stop 9 at instant 10. With this basis, we generate the first two rows’ and the last two rows’ timetable, where the interval between two vehicle departures is 5 min. More parts of the timetable can be generated in the same way, but they are not shown. As the timetable T only provides the information about the time instants at stops rather than the positions between two stops, the time instants at which the vehicles pass the nearest points to customers need to be estimated. For this, we assume the vehicles run at constant speeds between any two neighbour stops. Other system parameters are: $E_{0}=32$ unit, ϵ=2 unit, p=q=1 unit per minute, and v=1 km per minute (The values for the parameters $E_{0}$, *p* and *v* are based on the data of the Amazon Prime Air project in which a drone can fly about 32 km in 30 min.).

**Figure 6:**
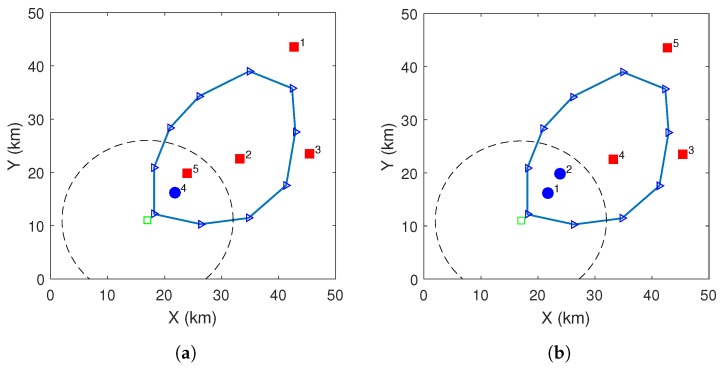
(**a**) Schedule by the exact algorithm. (**b**) Schedule by the sub-optimal algorithm.

Firstly, consider a case with 5 randomly generated customers, see Figure 6, where the schedules by the two algorithms are shown. The labels near the customers give their positions in the permutations. The delivery schemes are marked differently: red squares are by drone–vehicle scheme and blue circles are by drone-direct scheme. The optimal schedule delivers to one customer by the drone-direct scheme while the sub-optimal schedule delivers two customers by the drone-direct scheme. Their delivery permutations are different. The schedule by the exact algorithm uses 210 min to deliver to all the customers and the algorithm running time is about 0.333 s, while that of the sub-optimal algorithm uses 215 min, which is very close to the optimal solution, and it takes only 0.001 s to run the algorithm (The algorithm running time is measured in Matlab on a PC with: Intel(R) i5-3570S CPU and 8 GB RAM). For the comparison with the drone-direct only scheme, we also show the delivery area by this scheme (which is the black dash circle). For this example, only 2 out of the 5 customers can be delivered by the drone-direct scheme, while they all can be delivered by the proposed delivery system. This feature exists in all the other simulated cases. Of course, due to the randomness, there are some customers that cannot even be delivered by the drone–vehicle scheme, but the deliverable ratio of the drone–vehicle scheme is generally larger than that of the drone-direct scheme.

**Figure 7:**
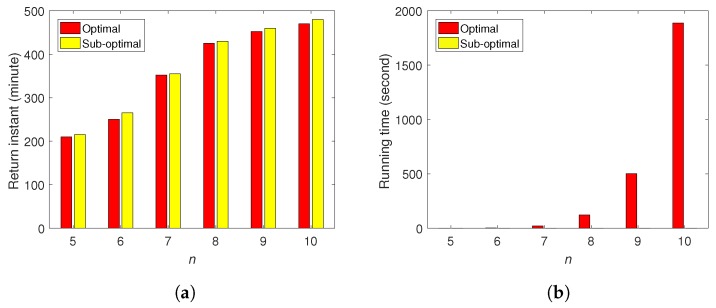
(**a**) Return instants for different n. (**b**) Algorithm running time. The results of the sub-optimal algorithm are all lower than 0.1 s.

We conduct more simulations for different numbers of customers *n* to demonstrate the performance of the sub-optimal algorithm against the exact algorithm. As the complexity of the exact algorithm is exponential to *n*, we only conduct simulations with up to 10 customers using the exact algorithm. As shown in Figure 7a, in all these simulations, the return instants by the sub-optimal algorithm are only 5 to 15 min more than the schedules obtained by the exact algorithm. The advantage of the sub-optimal algorithm lies in the computational efficiency. As shown in Figure 7b, the algorithm running time of the exact algorithm increases dramatically with *n*. However, that of the sub-optimal algorithm is lower than 0.1 s for these cases. From these results we can see that the proposed sup-optimal algorithm achieves close performance to the optimal solution and it is computationally efficient.

Moreover, we investigate the influence of some key parameters on the sub-optimal algorithm. Here, we consider large scale cases where the number of customers can be 20, 30, and even more. The customer locations are again randomly generated and the sub-optimal can address the case with 30 customers in seconds.

**Figure 8:**
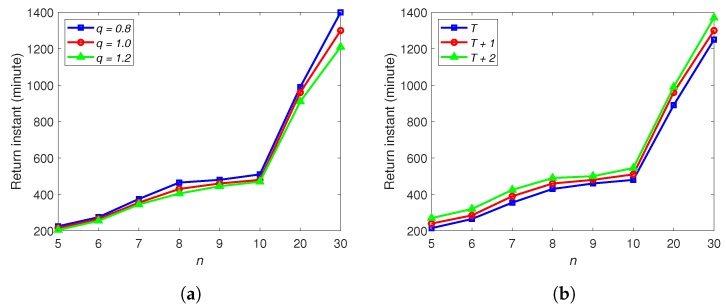
(**a**) The influence of *q*. (**b**) The influence of T.

We first consider the parameters of *p* and *q*. To study their impacts, we fix *p* as 1 and consider some values of *q*. When q<1, the recharging time becomes longer to recharge a certain amount of energy than the consumption of the same amount; while when q>1, the recharging process becomes faster. It can be seen from Figure 8a compared to the cases with q=1, when q=1.2, the return instants for the above cases become smaller; while when *q* is decreased to 0.8, the return instants all become larger. Clearly, the larger the *q*, the more efficient in terms of recharging, and then the drone can be back to deliver the next customer sooner.

The timetable T also has a significant impact on the scheduling performance. We create two more timetables: T+1 and T+2. Based on T, we increase the time interval between two neighbour stops by 1 min and 2 min, respectively, to get T+1 and T+2. As seen in Figure 8b, with T+2, the drone uses the longest time to complete the deliveries, which is followed by the timetable T+1. In other words, a longer interval between stops worsens the time for delivery, because it takes a longer time to travel with the vehicles if the drone–vehicle scheme is selected.

**Figure 9:**
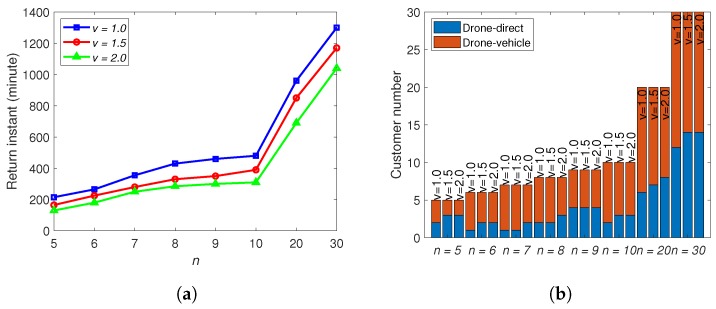
(**a**) The influence of *v*. (**b**) The number of customers served by different delivery schemes for different *v*.

Finally, the impact of the drone speed *v* is considered. In all the above simulations, the value of *v* together with those of $E_{0}$ and *p* are consistent with Amazon Prime Air project. Here, we keep the values for $E_{0}$ and *p*, and consider some higher speed. The timetable T is used here. According to our model, increasing *v* enlarges the delivery area of both the drone-direct scheme and drone–vehicle scheme, see (3) and (9). The final return instants for all the cases have a decreasing trend, see Figure 9a, with the increase of *v*. However, when *v* doubles, the new return instants are not half of the old values. The reason lies in the utility of the vehicles, whose travel times are not impacted by *v*. Moreover, when *v* increases, the delivery areas of these two schemes are enlarged. Then, the delivery scheme selection will be different. As shown in Figure 9b, the ratio of using drone-direct scheme has an increasing trend with the increase of *v*, because higher speed not only increases the number of deliverable customers but also decreases the trip time, which is much more significant for the drone-direct scheme than the drone–vehicle scheme.

For summary, in this section, we have shown that the sup-optimal achieves close performance to the exact algorithm and the former is much more computationally efficient than the latter. The study of the parameter turnings presents how these parameters impact the system performance.

## 6. Conclusions and Future Work

In this paper, a novel parcel delivery system consisting of a drone and public transportation vehicles was proposed. It involves the drone-direct scheme and the drone–vehicle scheme. The fundamental characteristics such as the trip time, energy consumption and battery recharging were modelled. A time-dependent scheduling problem for a single drone was formulated, which is NP-complete. An exact algorithm was first presented but it is computationally inefficient. Then, a sub-optimal solution was developed, which achieves close performance to the exact algorithm and is much more computationally efficient than the exact algorithm. The study of the parameter turnings presents how these parameters impact on the system performance. One future research direction is to replace the vehicles with a simple route by a more complex public transportation network. This definitely enlarges the delivery area but also brings some challenges in the estimations of trip time, since the transportation network is with more uncertainty. Another direction is to develop algorithms for multiple drones. The third is to extend the static scenario where all the orders are ready before the scheduling to the stochastic scenario where customers place orders in a dynamic manner. The latter case will be useful in real applications where suppliers wish to service the dynamic orders in the shortest time.

## Figures and Tables

**Table 1:** Notations and descriptions.

Notation	Description
R	Vehicle route
T	Vehicle timetable
S	A set of stops on R
*v*	Drone speed
*p*	Energy consumption rate
*q*	Battery recharging rate
$E_{0}$	Battery capacity
ϵ	Safety residual energy
$\epsilon_{1}$	Energy consumption for take-off
$\epsilon_{2}$	Energy consumption for landing
$\delta_{1}$	Time needed for take-off
$\delta_{2}$	Time needed for landing
$\tau_{r}\left(c\right)$	Charging time for delivering customer *c*
$\tau_{t}\left(c\right)$	Trip time for delivering customer *c*
$\tau_{f}\left(c\right)$	Flight time for delivering customer *c*
$t_{d}\left(c\right)$	Departure instant for delivering customer *c*
$t_{r}\left(c\right)$	Returning instant after delivering customer *c*
$d_{d}\left(c\right)$	Distance between customer *c* and the depot
p(c)	The closest point on R to customer *c*
$d_{r}\left(c\right)$	Distance between p(c) and customer *c*
s(c)	The closest stop among S to customer *c*
$d_{s}\left(c\right)$	Distance between s(c) and customer *c*
